# Quality improvement in healthcare: the need for valid, reliable and efficient methods and indicators

**DOI:** 10.1093/intqhc/mzz077

**Published:** 2019-11-08

**Authors:** Mohaimenul Islam, Yu-Chuan (Jack) Li

**Affiliations:** 1 Graduate Institute of Biomedical Informatics, College of Medicine Science and Technology, Taipei Medical University, 250-Wuxing Street, Xinyi District, Taipei, Taiwan; 2 International Center for Health Information Technology (ICHIT), Taipei Medical University, 250-Wuxing Street, Xinyi District, Taipei, Taiwan; 3 Department of Dermatology, Wan Fang Hospital, No. 111, Section 3, Xinglong Road, Wenshan, Taipei, Taiwan

Quality of care and patient’s safety are now recognized globally as a healthcare priority. While adverse events (AEs) are a serious issue related to the patient’s safety, concern has been raised on the quality of care provided globally. It is reported that AEs reckon additional 13–16% costs alone due to only prolonged hospital stay. The annual cost of prolonging hospital stay because of AEs is ~£2 billion in the UK [1]. Moreover, other issues like pain and suffering, loss of independence and productivity of patients or costs of litigation and settlement of medical negligence claims are often ignored while calculating the total economic burden of AEs. An increased number of AEs always have detrimental effects on both patients and healthcare providers including physical and mental harm, reducing credibility of the healthcare system. It is therefore important to identify and measure AEs for prioritizing problems to work on and making sophisticated ideas for better patient care as they generate substantial burden to patients and healthcare providers [2]. Although there is no gold standard for measuring AEs, a significant number of studies used the Harvard Medical Practice Study (HMPS) approach as a standard methodology for measuring AEs [3]. Trigger tools like the global trigger tool (GTT) (introduced by the institute for healthcare improvement) have been developed to identify and measure the AEs. It is an easy and less labor intensive two-stage method of retrospectively manual review of the patient’s chart. Firstly, two nurses individually screen patients’ reports for specific triggers and ascertain AEs regarding these triggers before making any decision. Secondly, physicians verify them based on the standard definition [4].

A study based on the local analysis at hospital reported that the GTT method had both high sensitivity and high specificity than other methodologies like HMPS [5]. Moreover, the GTT method correctly detected most of the AEs that were missed out by the Agency for Healthcare Research and Quality’s Patients Safety Indicators. Mevik *et al*. [6] evaluated a modified GTT method with a manual review of automatically triggered records to measure AEs using the original GTT method as a gold standard. However, the modified GTT method was more reliable and efficient when it came to monitoring and accurately identifying AEs. While comparing time to identify AEs, modified GTT took less time than original GTT (a total of 23 h to complete the manual review of 658 automatic triggered records with modified GTT compared to 411 h of review of 1233 records with the original GTT) but both of the methods identified same amount AEs (35 AEs) per 1000 patient’s days. Modified GTT would be the right choice for the higher sample size as it provides an effective alternative to valid, efficient and time-consuming approaches to identify and monitor AEs. In the future, an automatic trigger-identifying system with electronic health records might enhance the utility and assess triggers in real time to lessen the risk of AEs.

The health of pregnant women and child still remains a serious public health issue. Despite comprehensive efforts and investments in the healthcare sector; maternal and child mortality is unacceptably high [7]. Antenatal care (ANC) is of paramount importance for ensuring optimal care of pregnant women and reducing the risk of stillbirths and neonatal deaths. Quality of care during pregnancy and childbirth can help to reduce pregnancy complications and improve the survivals and health of babies. According to the updated recommendation by WHO, women need first ANC visit within the first trimester and an additional seven visits are recommended [8]. However, raised awareness, trained healthcare workers, strong national information and surveillance systems are needed for proper monitoring and timely and respectful care.

Morón-Duarte *et al*. [9] conducted a systematic review to describe indicators used for the assessment of ANC quality globally under the WHO framework. A total of 86 original studies were included, which described the ANC model and ANC quality indicators such as the use of services, clinical or laboratory diagnostic procedure and educational and prophylactic intervention. The quality of the included studies was evaluated according to the ‘Checklist for Measuring Quality’ proposed by Downs and Black [10]. A highly diverse and region-specific description of indicators was observed while relevance and use depend on the country-specific context. However, 8.7% of the articles reported healthy eating counseling and 52.2% iron and folic acid supplementation on the basis of updated WHO recommendation. The evaluation indicators on maternal and fetal interventions were syphilis testing (55.1%), HIV testing (47.8%), gestational diabetes mellitus screening (40.6%) and ultrasound (27.5%). Essential ANC activity assessment ranged from 26.1% report of fetal heart sound, 50.7% of maternal weight and 63.8% of blood pressure. Concern has been raised due to the quality assessment of ANC content especially in the utilization of services across countries. It is important to use health indicators based on the international guidelines but the appropriateness of suggested indicators and the construction of structured and standardized indices are necessary to be implemented in the different countries allowing international comparability and monitoring.

In summary, various trigger tools have been implemented and evaluated to improve drug therapy assessment and monitoring in hospitalized patients, nutrition support practice and tertiary care hospitals, but these tools need to be validated in varied patients’ populations. Moreover, improved quality of care and patient’s safety initiatives are essential to reduce AEs and maternal and newborn mortality.
